# Quantitative assessment of *Azumiobodo hoyamushi* distribution in the tunic of soft tunic syndrome–affected ascidian *Halocynthia roretzi* using real-time polymerase chain reaction

**DOI:** 10.1186/s13071-014-0539-x

**Published:** 2014-11-26

**Authors:** Yun-Kyung Shin, Ki-Woong Nam, Kwan Ha Park, Jong-Man Yoon, Kyung-Il Park

**Affiliations:** National Fisheries Research and Development Institute, Busan, 619-705 Republic of Korea; Department of Aquatic Life Medicine, College of Ocean Science and Technology, Kunsan National University, 558 Daehakno, Gunsan, 573-701 Republic of Korea

**Keywords:** *Halocynthia roretzi*, *Azumiobodo hoyamushi*, Real-time PCR, Soft tunic syndrome

## Abstract

**Background:**

The kinetoplastid parasite, *Azumiobodo hoyamushi*, is the causative agent of soft tunic syndrome (STS) in ascidians and leads to their mass mortality in Korean waters. This study was conducted to quantify *A. hoyamushi* density during the development of STS in the tunics of ascidians (*Halocynthia roretzi*) using real-time polymerase chain reaction (qPCR).

**Findings:**

The infection intensity of *A. hoyamushi*, as measured by qPCR, varied depending on the part of the tunic analyzed, as well as the stage of STS development. The highest infection intensity was recorded in the tunics of the siphons. The infection intensity of *A. hoyamushi* in the siphons was only 2.9 cell/tunic (area, 0.25 cm^2^) or 106.0 cell/gram tunic (GT) in the early phase of STS, but this value increased dramatically to 16,066 cells/tunic (0.25 cm^2^) or 617,004 cell/GT at the time of death. The number of *A. hoyamushi* parasites increased gradually and their distribution spread from the siphons to the other parts of the tunics.

**Conclusions:**

qPCR enabled the quantitation of *A. hoyamushi* and the results revealed that parasite density increased as STS progressed. In addition, our results suggested that the siphons might function as the portal of entry for *A. hoyamushi* during infection.

## Background

Ascidians (*Halocynthia roretzi*) belong to class Ascidiacea, order Pleurogona, and family Pyuridae, and have been commercially important marine invertebrates in Korea for decades [1]. However, over the past 20 years, soft tunic syndrome (STS) has been plaguing the Gyeongnam Province area on the southern coast of Korea, which has most of the country’s ascidian farms, causing mass mortality of the ascidians. In Japan, outbreaks of ascidian STS have been reported in farms located in the Miyagi Prefecture since 2007 [2].

A flagellated protozoan was isolated from an ascidian affected with STS, and the flagellate was subsequently identified as *Azumiobodo hoyamushi* by Japanese investigators [3,4]. Recently, our group also isolated a kinetoplastid protozoan from STS-affected ascidians cultured in the East Sea of Korea and found that the 18S rRNA sequence of the organism was highly homologous to that of the *A. hoyamushi* isolated in Japan, suggesting a common causative pathogen for STS in both Korea and Japan [5].

STS causes the tunic fiber bundle to disintegrate by diminishing the rigidity and integrity of the tunic fiber. Due to low fiber density, the tunics become thin and soft and finally rupture leading to leakage of internal tissues and death [6-9]. Breakdown of the tunics is mediated by a metalloprotease enzyme secreted by *A. hoyamushi* [10].

Diagnostic methods for the pathogen include histology and conventional polymerase chain reaction (PCR), and these tools have helped understand STS pathogenesis [11,12]. A couple of reports have suggested that the siphons are the possible entry routes for the parasites in the ascidians, although the disease is not easily identifiable until apparent symptomatic changes occur in the central tunic of the STS ascidians [2,13]. According to this hypothesis, if the parasite initially infects the tissues of the siphon and then subsequently propagates to the central tissues, there will be a sequential distinction in the number of parasites at different tunic tissues of the same symptomatic stage. It is also likely that progressively higher parasite numbers will be observed at the same site as the severity of symptom heightens. In this manner, parasite numbers will be closely correlated with the progression of soft syndrome in a given tunic tissue. This study was designed to evaluate the severity of the syndrome at different tunic sites with enhanced quantitative accuracy, employing qPCR for parasite enumeration. This experimental design will help understand the anatomical progression of the disease. Additionally, the study design might aid early parasite detection in the ascidians. Furthermore, to our knowledge, no study has examined the changes in parasite numbers in different portions of the diseased tunic.

## Findings and discussion

**Figure 1:**
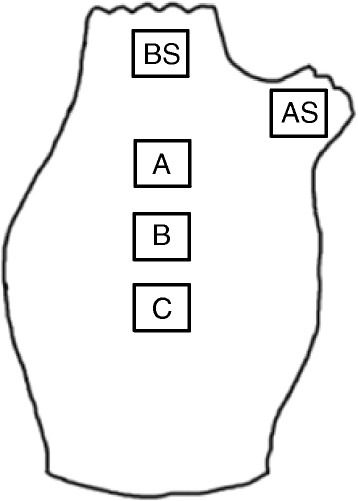
**A diagram illustrating the parts of the ascidian tunic from where tissues were excised and used for qPCR.**

The ascidians used in this study were farm-raised in Tongyeong on the southern coast of Korea, where STS is endemic. Ascidians were transported to a laboratory tank that contained filtered seawater (filtered using a 2-μm filter, 30 psu). The ascidians were kept in seawater at 15°C for 2 weeks. During this period, the various phases of STS were developed, and the STS developmental phase was classified according to Kitamura’s scale (KS) [11]. Three ascidians in each KS phase were selected, and tunic tissues (area, 0.25 cm^2^) from the atrial siphon, branchial siphon, and 3 other parts of the tunic were excised from each ascidian and weighed (Figure 1). Then, DNA was extracted using a DNA extraction kit (Qiagen, USA) and eluted in 200 μL of Buffer AE (Qiagen, USA). qPCR was performed in triplicate on the extracted DNA as described below.

**Figure 2:**
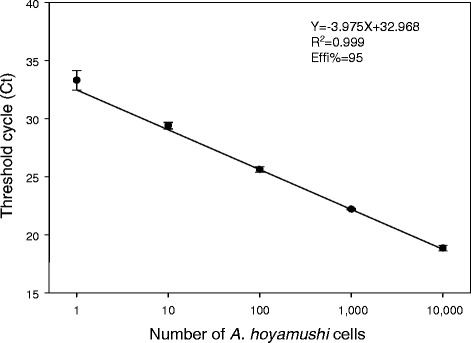
**Plot of mean Ct values against the logarithmic quantity of** ***Azumiobodo hoyamushi*** **DNA (1 to 10,000 cells per reaction).**

To construct a standard curve for the quantification procedures, *A. hoyamushi* was cultured *in vitro* according to our previous study [5]. DNA was extracted from 1,00,000 cells cultivated *in vitro* using a DNA extraction kit (Qiagen, USA), and the DNA was eluted in 200 μL of Buffer AE (Qiagen, USA). Triplicate serial 10-fold dilutions were performed to obtain DNA concentrations corresponding to 10,000, 1,000, 100, 10, and 1 cell(s). For the qPCR, 17 μL of extracted DNA, 10 pmol of forward primer (1 μL; 5′-TGA GCG TGA GAG GTG AAA-3′), 10 pmol of reverse primer (1 μL; 5′-CTC AAT CAA GAA CCA AAG TGT G-3′), and 10 pmol of TaqMan probe (1 μL; 5′ FAM-CCG CTC AAA GAC GAA CTA CAG CGA-BHQ1 3′) were added to a premix (AccuPower® DualStar™, Bioneer, Korea). The primer pair and the probe were designed based on specific regions in the 18S rRNA of *A. hoyamushi* isolated from the ascidians of Tongeong, Korea (GenBank accession number, KF479402), and DNA extracted from the tunic of an STS-free ascidian and kinetoplastid protozoans (*Procryptobia sorokini*) were used as the negative controls. The STS-free ascidian was obtained by the formalin bath method [14], and *P. sorokini* was isolated from STS-affected ascidians and propagated *in vitro* in minimum essential medium [5]. The cycling program consisted of an initial denaturation step at 94°C for 5 min, followed by 45 cycles each of 94°C for 15 s, 47°C for 1 min and 72°C for 30 s in a qPCR machine (Exicycler™, Bioneer, Korea). The primers and probes did not cross-react with the DNA from the STS-free ascidian (*H. roretzi*) or the kinetoplastid protozoan (*P. sorokini*) isolated from STS-affected ascidians (data not shown). The correlation coefficient, obtained from a linear regression analysis, was *R*^2^ = 0.999, with a slope of −3.975 and an intercept of 32.968 (Figure 2). Efficiency, as determined from the slope and using the formula *E* =10^−1/slope^ −1, was 95%. The real-time probe could detect *A. hoyamushi* with high sensitivity (as few as 1 cell/mL).

**Figure 3:**
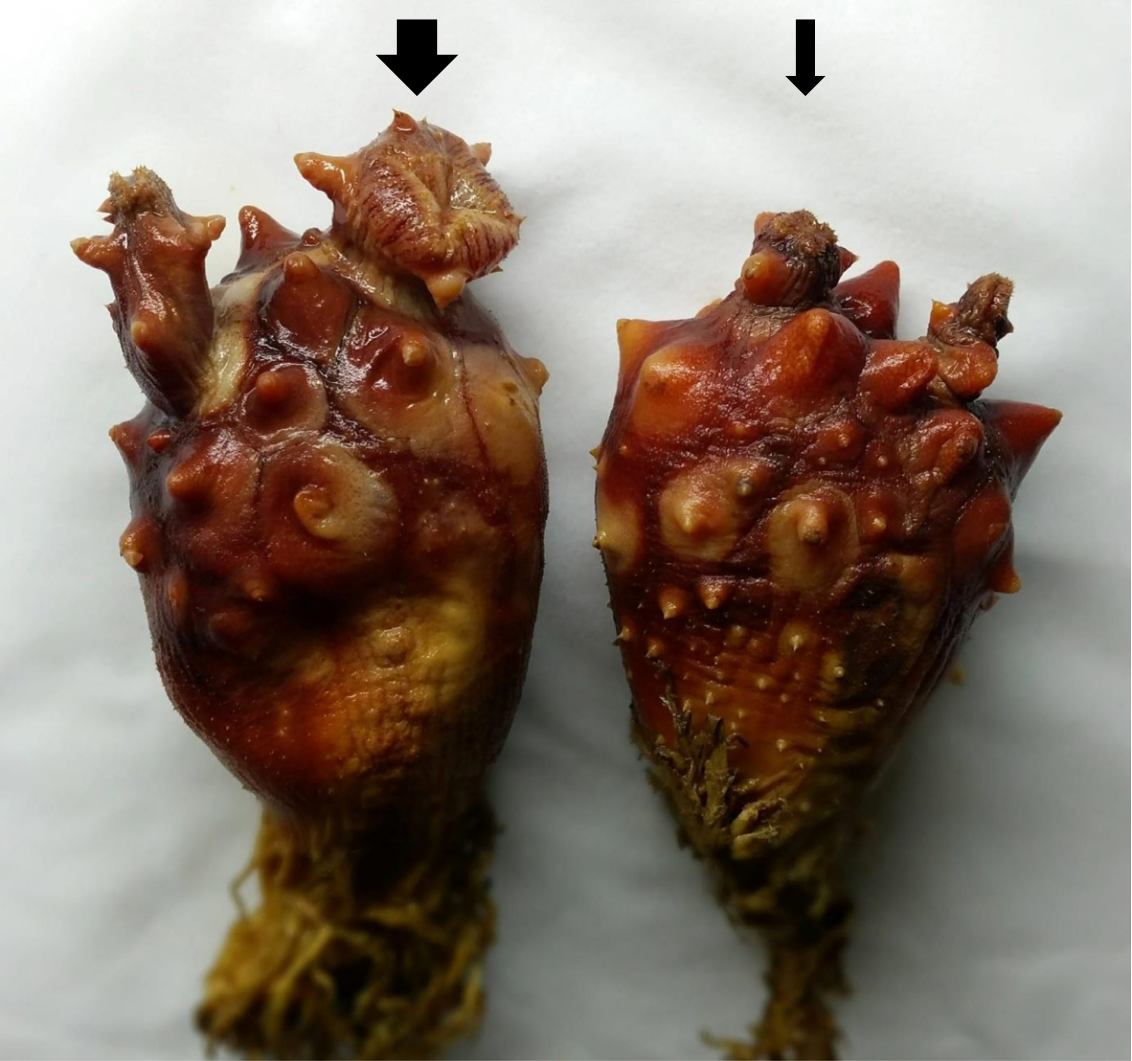
**Swelling (large arrow) and normal (small arrow) of the branchial and atrial siphons of** ***Halocynthia roretzi*** **caused by** ***A. hoyamushi*** **infection.**

Subsequently, the PCR was used to quantify the parasites in various tunic locations in different STS developmental phases. Although the ascidians were classified as KS-1 (early phase of STS with no external STS symptoms), the density of *A. hoyamushi* in the atrial and branchial tunics of ascidians was 2.9 cell/tunic (area, 0.25 cm^2^) or 106.0 cell/gram tunic (GT). This increased dramatically to 160.1 (KS-2), 2,994.6 (KS-3), and 16,066.9 cells/tunic (0.25 cm^2^, KS-4 phase) or 7,939.6, 39,093.3, and 617,004.1 cell/GT, showing the highest infection intensity among the various parts of the ascidian tunic examined. As STS progressed, higher densities of *A. hoyamushi* were found in other parts of the tunics as well (Table 1). Such high infection intensity in the siphons in the early phase of *A. hoyamushi* infection suggested that the siphons are probably a portal of *A. hoyamushi* invasion. This finding supports the hypothesis proposed in a previous report [13]. Indeed, swollen siphons are easily recognizable in the early phase of STS, and our study suggests that these irregular siphons might be a pathologic symptom of STS (Figure 3). Recently, a damaged portion in the cuticle layer situated in the inner surface of the siphon was observed, and it was suggested that this damaged region in the siphon wall might serve as the entry route for *A. hoyamushi* during the first stage of invasion [13]. This explains the considerably higher infection intensity in the siphons compared with that in the other parts of the tunic during the early phase of infection (KS-1). In addition, it is believed that the siphons are most suited for *A. hoyamushi* detection because of the presence of high infection intensity.

**Table 1:** **The number of** ***A. hoyamushi*** **in tunics in various KS phases, as measured using qPCR**

**Unit**	**Tunic**	**Kitamura's scale**			
		**1**	**2**	**3**	**4**
# of *A. hoyamushi/*0.25cm^2^	BS	2.9 ± 0.4	160.1 ± 16.7	2,994.6 ± 1,783.5	16,066.9 ± 2,609.9
	AS	2.9 ± 0.6	46.6 ± 30.3	2,337.6 ± 424.1	4,121.0 ± 281.7
	A	0.2 ± 0.2	0.4 ± 0.5	1,912.9 ± 650.3	5,053.2 ± 2,560.8
	B	0.0 ± 0.0	38.0 ± 1.1	2,339.4 ± 257.5	5,599.2 ± 915.1
	C	0.2 ± 0.0	3.6 ± 0.0	1,362.0 ± 462.1	5,396.1 ± 507.3
# of *A. hoyamushi/*GT	BS	106.0 ± 1.3	7,939.6 ± 1,241.9	39,093.3 ± 18,334.2	617,004.1 ± 10,949.2
	AS	85.5 ± 1.8	3,012.5 ± 2,607.6	70,866.4 ± 6,961.5	131,835.5 ± 37,352.4
	A	6.0 ± 2.6	12.4 ± 13.7	49,108.0 ± 6,987.4	169,404.2 ± 94,002.7
	B	0.3 ± 0.4	1,005. 2 ± 44.8	59,569.7 ± 6,555.5	205,291.7 ± 24,037.0
	C	3.1 ± 0.6	114.9 ± 23.6	31,702.4 ± 7,770.8	211,555.4 ± 86,528.5

Kitamura's scale (KS)-1: ascidians with no soft tunic syndrome (STS) symptoms but collected from STS-affected population; KS-2: ascidians exhibiting mild STS; KS-3: ascidians with advanced STS; KS-4: seriously diseased ascidians with ruptured tunics.

BS: branchial siphon; AS: atrial siphon.

A high density of live *A. hoyamushi* was visible in the tunics from ascidians with KS-3 phase STS upon observation under a light microscope (data not shown). Currently, microscopic enumeration using a hemocytometer is commonly used to quantify *in vitro*-cultured *A. hoyamushi* [3]. However, because *A. hoyamushi* is present within the tunic fiber tissue, which consists of a rigid cellulose structure [2,3,15], accurate quantification of *A. hoyamushi* cannot be achieved using the simple microscopic method. Accordingly, qPCR can provide a more accurate method for the quantitative evaluation of *A. hoyamushi* in ascidian tunics.

In conclusion, we developed a method to quantify *A. hoyamushi* using qPCR and found that the density of *A. hoyamushi* varied from a few individuals to several thousands of the pathogen depending upon the phase of STS development. Our results also suggest that the siphons may serve as the portal of entry for *A. hoyamushi*. The quantification of *A. hoyamushi* in ascidians and their environments may provide more information about the etiology of STS and help predict and monitor outbreaks of STS in the future.
